# Leveraging metatranscriptomics for the characterisation of bovine blood viromes

**DOI:** 10.1038/s41598-025-20367-1

**Published:** 2025-10-21

**Authors:** Barbara Brito, Matthew DeMaere, Ian Lean, Mark Hazelton, Brendon A. O’Rourke, Edward C. Holmes, John K. House, Sam Rowe, Garry S. A. Myers, Piklu Roy Chowdhury

**Affiliations:** 1 Australian Institute for Microbiology and Infection, University of Technology Sydney, Ultimo, NSW Australia; 2 Department of Primary Industries and Regional Development (DPIRD), New South Wales, Elizabeth Macarthur Agricultural Institute, Menangle, NSW Australia; 3 Scibus, Camden, NSW 2570 Australia; 4 School of Medical Sciences, The University of Sydney, NSW Sydney, Australia; 5 Faculty of Science, Sydney School of Veterinary Science, The University of Sydney, Camden, NSW 2570 Australia

**Keywords:** Metagenomics, Bovine, Blood virome, Computational biology and bioinformatics, Diseases, Microbiology

## Abstract

Understanding the diversity of the bovine virome is essential for assessing their potential impact on cattle health and transmission risks. Viruses present in the blood comprise both those that establish persistent infections in blood cells and those present during transient viremia. Farm management practices, such as the reuse of syringes for treatments, vaccinations, and supplements, may inadvertently contribute to the spread of blood-borne pathogens, emphasizing the need for improved biosecurity measures. Herein, we used a metatranscriptomic approach to analyse 20 bovine blood transcriptomes from dairy cows in New South Wales, Australia, along with 577 publicly available blood transcriptomes from studies in Australia and Kenya. Our analysis identified several viruses that are known to infect blood cells, transmitted either by direct contact or by vectors, including bovine viral diarrhea virus, bovine gammaherpesvirus 6, hepacivirus, foamy virus, ephemeroviruses and a new species of a coltivirus. Our findings highlight the complexity of the bovine blood virome and underscore the importance of sustained surveillance to identify emerging pathogens and assess their potential role in cattle health. This study provides a framework for integrating transcriptomic data into disease monitoring efforts, ultimately contributing to improved cattle management and biosecurity practices.

**Supplementary Information:**

The online version contains supplementary material available at 10.1038/s41598-025-20367-1.

## Introduction

 Culture-independent untargeted sequencing approaches have enabled a deeper understanding of microbial communities through a comprehensive taxonomic profiling of clinical and environmental samples^1^. This approach has facilitated research that explores the intricate and dynamic interactions between microbes and their hosts across various biological systems. In ruminants, research has focused on systems such as the rumen, while other groups have incorporated untargeted tools in the study of enteric and respiratory health and disease^2–5^. However, other biological entities such as blood, also play significant roles in shaping host-microbe interactions, which can impact overall health, disease susceptibility, and productivity.

Traditionally, blood was assumed to be a sterile environment in healthy individuals. However, most of our current knowledge about blood-associated viruses comes from human studies, where culture-independent sequencing has revealed that viral and bacterial nucleic acids are commonly detectable even in asymptomatic individuals^6^. For example, in humans, Anelloviruses circular single stranded DNA viruses are dominant in the blood virome of healthy individuals, while different species of herpesviruses are also frequently detected across both healthy and diseased states^7^. Therefore, latent or persistent viral populations can be a common feature of blood. In humans, the global spread of blood-borne viral diseases has been profoundly shaped by medical and behavioural practices. Widespread transfusions, unscreened blood products, and syringe reuse, particularly before pathogen screening protocols were established, unintentionally facilitated transmission, contributing to enduring public health challenges^8,9^.

While the spread of blood-borne viruses in humans has often reflected unintended consequences of medical and social practices, growing attention is now being directed toward understanding how some dynamics, through biological products, management practices, and vector exposure, may shape the virome of livestock species such as cattle. Studies of the bovine blood, plasma, and serum virome have primarily used high-throughput or metagenomic sequencing to characterize viral diversity and detect potential contaminants in commercial bovine serum. Collectively, these studies aimed to better understand viral persistence in cattle, identify novel or unexpected viruses, and improve biosafety of biological products. Analyses of bovine serum and plasma have consistently reported a predominance of parvoviruses, together with diverse small circular ssDNA viruses (CRESS-DNA, including circoviruses and gemycircularviruses)^10–12^. Additional studies have detected bovine hepacivirus, pestiviruses, papillomaviruses, and foamy viruses, highlighting both known and novel viral taxa that can circulate in blood, and in some cases even enteric viruses such as noroviruses and kobuviruses^10,11,13^. In addition to viruses, there is a high prevalence of bacterial pathogens such as *Anaplasma* spp., *Mycoplasma* spp. (notably haemoplasmas) detected using targeted approaches^14,15^, as well as vector-borne protozoan pathogens like *Babesia* and *Theileria* in regions where the tick vector is endemic^16,17^.

In livestock, both inadvertent transmission through management practices and natural transmission, such as changes in the distribution of capable hematophagous arthropods, influence the incidence and prevalence of blood-borne diseases within herds^18^. Unintentional transmission through the repeated use of syringes for treatments, vaccinations, or supplements, along with practices such as ear tagging and inadequate sterilization of equipment used in procedures like castration, may also contribute to blood-borne pathogen spread^19–21^.

In this study, we used a metatranscriptomic (i.e., total RNA sequencing) approach to analyse bovine blood transcriptomes, with the aim of profiling the blood virome in a defined set of samples from different study settings. By integrating publicly available bovine blood transcriptomic datasets with those generated by our research group, we sought to detect and characterise viruses present in the sampled animals. While several of the identified viruses are known to occur in cattle, our findings demonstrate the value of leveraging transcriptomic data to generate an untargeted and comprehensive view of the bovine blood virome. This approach can complement traditional surveillance by capturing both expected and potentially overlooked pathogens within existing datasets.

## Methods

The data sets analysed for this study originated from two sources: (i) Data set 1 -- a pilot study conducted by our research group on cows with and without mastitis, and (ii) Data set 2 -- publicly available datasets of cow blood transcriptomes. These datasets were originally collected for purposes unrelated to this study.

### Data set 1

#### Selection of animals, blood sampling methods and ethics

Blood samples were collected in March 2021 from Holstein cattle at a dairy farm in New South Wales (NSW), Australia, where animals were managed under a total mixed ration feeding system. While most replacement heifers were sourced from within the same farm, some had been purchased from external farms prior to sample collection. These samples were obtained for a pilot project investigating bovine markers of mastitis in blood. The samples included 35 cows diagnosed with mastitis and 35 healthy cows, identified as BM_1-BM_70. Blood was collected via coccygeal vein puncture into 10 mL PAXgene^®^ Blood RNA Tube (BD Biosciences) to stabilize RNA and then stored in a −80 °C freezer. Ethical approval for sampling was obtained following the institutional animal care guidelines at the Elizabeth Macarthur Agricultural Institute [AEC Reference: M20/6] in Menangle, NSW, Australia.

### RNA extraction

Two aliquots of 2.5 mL of blood samples were collected per cow and preserved in PAXgene^®^ Blood RNA Tubes following the manufacturer’s instruction. RNA was isolated using TRIzol reagent (Thermo Fisher Scientific, USA). Briefly, blood from the PAXgene tubes was thawed and stabilised at room temperature for 6 h, transferred to a 50 mL centrifuge tube and centrifuged at 4000 *x g* for 10 min at room temperature. The pellet was re-suspended in 1 mL TRIzol reagent and homogenised with repetitive pipetting. Two hundred microlitres of chloroform was added and shaken vigorously for 30 s and incubated at room temperature for 10 min. Following centrifugation at 12,000 *x g* for 15 min at 4 °C, the upper aqueous phase was transferred to a fresh 1.5 mL centrifuge tube. An equal volume of isopropanol was added, mixed and incubated on ice for 15 min. The aqueous and organic phases were separated by centrifugation at 12,000 *x g* for 15 min at 4 °C, and the top aqueous phase was washed with 75% ethanol. RNA was pelleted by centrifugation at 9,000 *x g* for 15 min at 4 °C and resuspended in nuclease-free water at room temperature, treated with TURBO DNase (Invitrogen USA) to remove genomic DNA contamination, following the manufacturer’s protocol. The RNA was purified using the RNeasy Mini Kit (Qiagen, Germany). The quality of the RNA was assessed on TapeStation and BioAnalyzer (Agilent, USA) to ensure all RNA samples met in-house quality control (QC) criteria (> 150ng of RNA and RIN value of 7). RNA aliquots were then sent to the Australian Genome Research Facility (AGRF), Melbourne, Australia, for whole transcriptome sequencing.

### Total RNA sequencing

Samples underwent further QC at the AGRF. Samples with a Distribution Value (DV) 200 score > 55 (percentage of RNA fragments > 200 nucleotides) were sent for sequencing library preparation using the Illumina Stranded Total RNA Prep and the RiboZero Plus kit was used to deplete mammalian rRNA. Of the 70 samples processed, 20 met the QC criteria for sequencing. The libraries were multiplexed and sequenced on the NovaSeq 6000 platform using 300-cycle runs, generating a total of 387.8 GB of sequencing data. Dataset 1 is available at NCBI BioProject PRJNA1250162.

### Data set 2

#### Transcriptomic data from public databases

In addition to the experimental data generated in-house, publicly available RNA-seq data sets were obtained from the NCBI Sequence Read Archive (SRA). Data were downloaded from three relevant NCBI BioProjects: (1) PRJEB44244: “Longitudinal study of blood-derived transcriptomes of Boran cattle naturally exposed to *Theileria parva*”^22^. This study included blood samples from 30 cows in Kenya in 2018 and RNA that was extracted from white blood cells at different time points, (2) PRJNA917329/PRJNA305942/PRJNA392196: “Gene expression and RNA splicing explain large proportions of the heritability for complex traits in cattle”, and comprised blood samples from 382 lactating cows from Australia^23^, and (3) PRJNA616134 **“**Gene expression of the heat stress response in bovine peripheral white blood cells and milk somatic cells in vivo” and included blood transcriptomes of 12 animals, also from Australia^24^. The samples from NCBI BioProjects PRJEB44244 and PRJNA616134 comprised transcriptomes from bovine white blood cells (WBC) in contrast to whole blood transcriptome obtained from this study. Details of sample collection, RNA extraction and library preparation of the published BioProjects are shown in Table S1.

### Detection of viral reads

Low-quality sequence reads and bases were removed using bbduk (BBTools 38.87)^25^. The number of reads and bases retained after QC are presented in Table S2. Trimmed reads were assembled *de novo* using SPAdes v3.13.0 with the –metaspades option.

^26^. Assembled contigs were classified using BLASTn against the NCBI viral nucleotide (nt) database. Bacteriophages and plant or fungal viruses were excluded from further analysis. Contigs mapping > 80% to non-viral genomes (e.g. host or bacterial) were flagged false positive and removed. Viral contigs were re-analysed against the NCBI nt database to rule out false positives. Only viral contigs from families known to infect mammals or arthropods were retained.

### Quantification of viral contigs

Host reads were removed using Bowtie2 aligned with Hostile wrapper v2.0.0, masking bacterial reads^27^. To approximate estimates of viral abundance, RNA reads from bovine transcriptomes were mapped against a multifasta reference file containing known bovine viruses and viral genomes identified from this study using kallisto 0.46.0 alignment program^28^. A sample was considered positive for a virus if five or more reads were mapped to the corresponding viral genome.

### Phylogenetic analysis

Identified viral contigs were aligned with the closest published references using MAFFT v7.525^29^. For divergent viruses (i.e., putative new virus species) we used the alignment of translated amino acid proteins. For the phylogeny of RNA viruses encoding polyproteins and from which near-complete genome was obtained, a recombination analysis using RDP4 was used to ensure phylogenies were estimated using recombinant free sequences^30^. Phylogenetic trees were estimated using the maximum likelihood method available in IQ-TREE using the -m TEST and -bb 1000 options^29,31^.

The resultant trees were visualized using iTOL^32^ and genetic distance plots were generated using the ape, gglot2, Biostrings and ggcoverages R packages^33–36^. The K80 nucleotide substitution model was applied with a sliding window size of 50 and a step size of 25 for the genetic distance plot.

Genome annotations were manually curated in Geneious Prime 2025.0.3 by transferring data from the closest annotated reference. Newly identified viral sequences were submitted to GenBank under accession numbers PV578941-PV578943 and BK071684-96 (Supplementary Table S3).

## Results

For data set 1, the average read length per sample was 141.31 bases, and following quality control and trimming the library sizes ranged from 99.99 M to 162.89 M reads. For data set 2, a total of 577 published transcriptomes were downloaded and analysed for this study.

### Viruses identified in the transcriptomes

#### Bovine hepacivirus

**Fig. 1:**
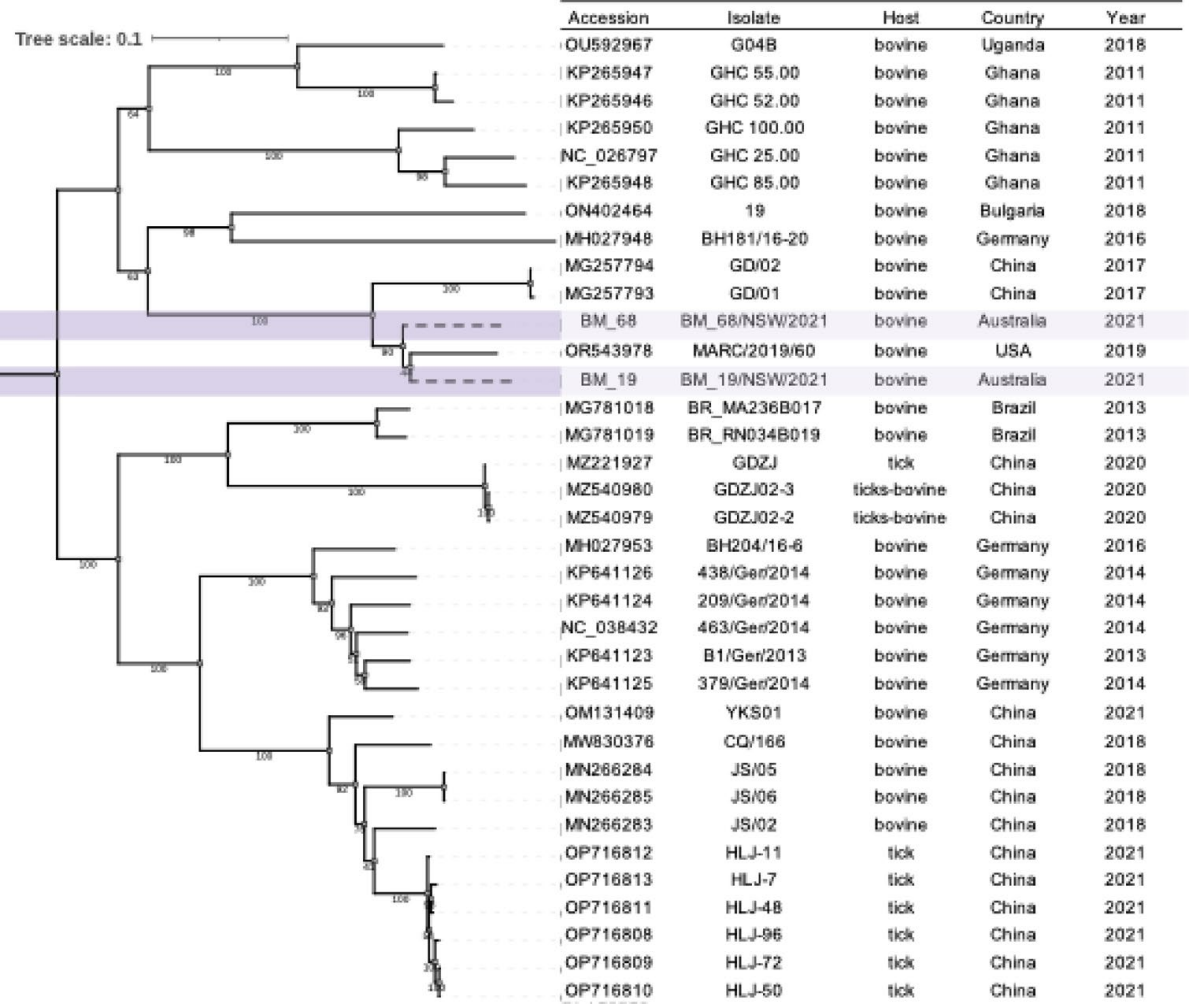
Maximum likelihood phylogeny (midpoint rooted; best substitution model selected: TIM2 + F + I + G4) of Bovine hepacivirus in the recombination-free coding region (s1; position in the alignment 898–4025). Metadata extracted indicates the host, country and year of collection of the samples. Shaded branches indicate the group that includes the sequences assembled from dataset 1. The viruses identified in this study were grouped with hepaciviruses collected from bovine samples in Africa, Europe and North America. The sister lineage contains viruses collected from ticks and bovines.

Near-complete genomes for bovine hepacivirus were assembled from two samples (#68 and #19), with contig lengths of 8,823 and 7,732 nucleotides, respectively. Bovine hepacivirus is a positive-sense single-stranded RNA virus (+)ssRNA from the family *Flaviviridae* with a complete genome length of approximately 8.8 kb. Phylogenetic analysis revealed that the closest published sequence to that obtained here was from a bovine hepacivirus collected in the US (OR543978.1_MARC/2019/60, US 2019). The genomic nucleotide similarity between the two sequences obtained in this study was 0.89, while their distances from the US reference sequence were 0.89 and 0.9, respectively (Fig. 1). The phylogenetic analysis was performed using recombination-free regions (nucleotide positions 898–4025 (s1) and 5328–8618 (s2)), although the tree topology remained consistent in both regions.

Across samples in dataset 1, bovine hepacivirus was identified in seven samples, representing three cows with mastitis and four without mastitis. No hepacivirus sequences were detected in any of the published transcriptomes analysed in data set 2.

#### Bovine gammaherpesvirus 6

**Fig. 2:**
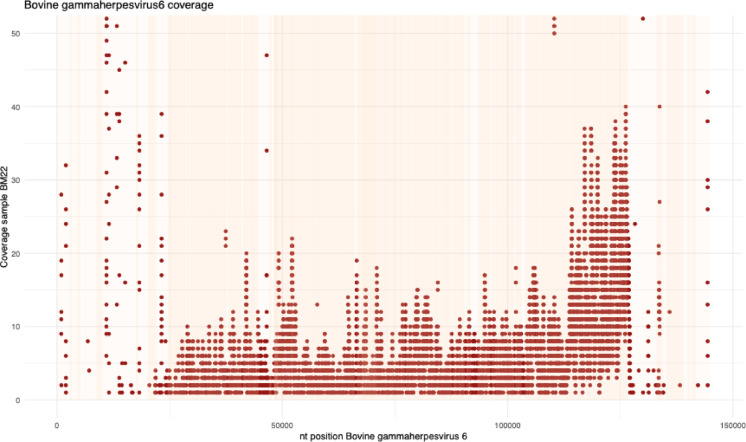
Contigs mapped to the bovine gammaherpesvirus-6 virus reference (NC_024303) from sample #22. The coverage of the contigs is spread throughout the genome.

A near-complete genome of bovine gammaherpesvirus 6 (BoGHV6) also known as bovine lymphotropic virus) was assembled from one sample from Data set 1 (#22). The genome sequence had a coverage breadth of 65.6% when the transcriptome was mapped to the reference NC_024303 (Fig. 2).

Sequence reads from one transcriptome from dataset 2 (Australian published transcriptome: BioSample: SAMN47932232) mapped to this virus. No reads from the Kenyan transcriptomes mapped to BoGHV6.

#### Pestivirus Bovis (Bovine viral diarrhea virus-1)

**Fig. 3:**
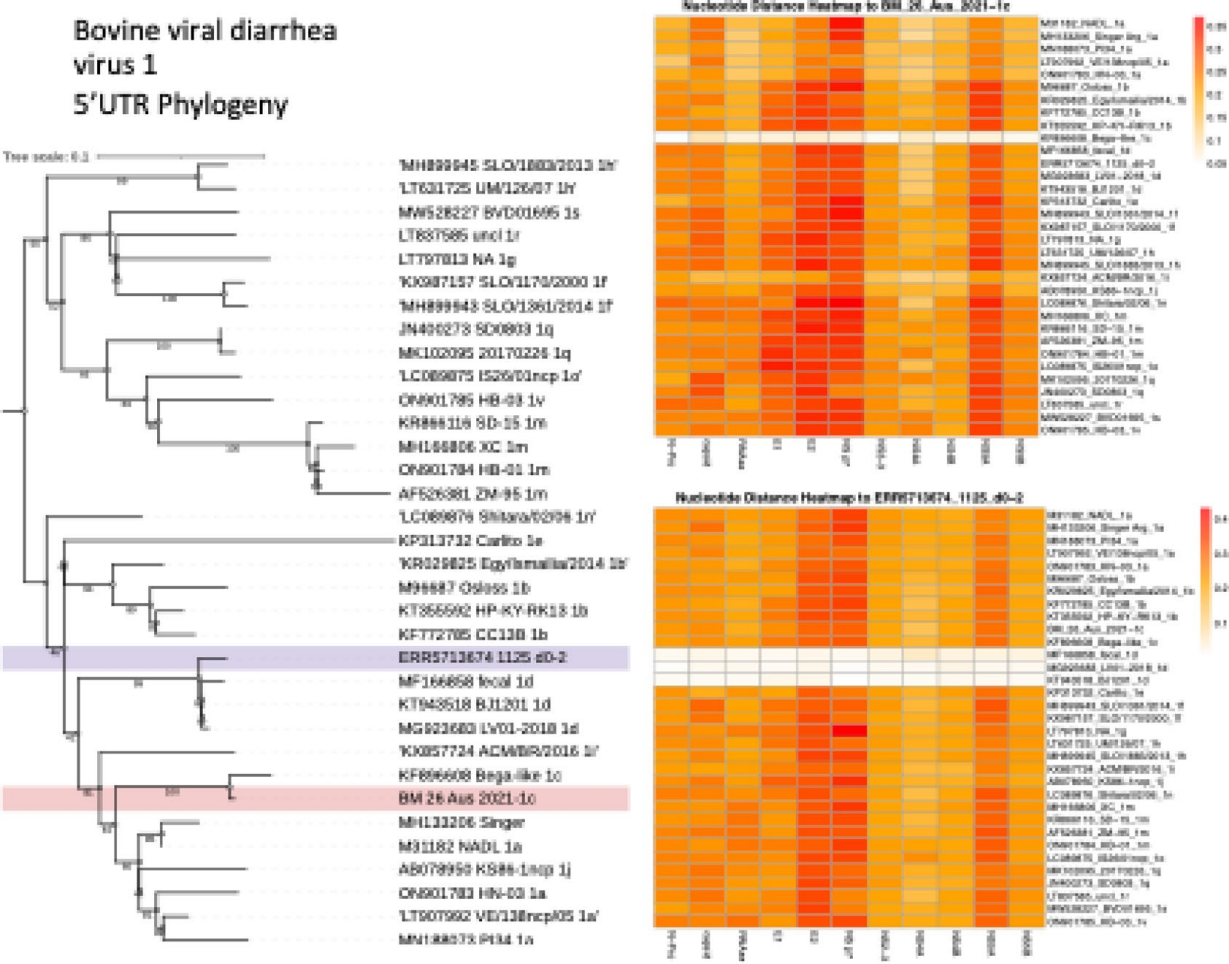
Phylogeny of 5’UTR (midpoint rooted) of sequences from viruses from all bovine viral diarrhea virus-1 subgenotype (left panel). A heatmap with nucleotide genetic distance (K80 substitution model) in each of the protein coding regions with reference viruses from different subgenotypes are shown for sample #26 from this study, with high similarity to other Pestivirus 1c (top right). Similarly, from the published library ERR5713674, within the clade of Pestivirus 1 d, all gene regions were closely related to viruses within the same sub genotype.

*Pestivirus bovis* was identified in one sample of data set 1 and one sample in data set 2. A near-complete genome BVDV 1c was assembled from a blood sample from a cow with mastitis (#26). The genome had a coverage depth of 232.3. From data set 2, a complete genome of BVDV 1 d was assembled from short reads of from Bioproject PRJEB44244 (SRA: ERR5713674) from blood collected from an Ankole cattle in Kenya. The virus was detected in multiple samples collected from a single animal on study days 0, 7 and 15. *Pestivirus bovis* belongs to the family *Flaviviridae*. Sequence comparison revealed that BVDV 1c had 94.88% identity to a reference sequence from China PQ782224.1, and a similar identity (94.03%) to the Bega-like BVDV 1c Australian reference strain. The closest match of the sequence found in data set 2 was a BVDV 1 d Pestivirus from a Holstein cow in China with 98.32% identity across the complete genome (KT951841.1, 2013; Fig. 3). The phylogenetic analysis of the 5′UTR region grouped the sequences from this study with their respective subgenotypes, 1c and 1d. Consistently, nucleotide similarity across all other protein-coding regions showed the highest identity to the same subgenotypes defined by the 5′UTR phylogeny (Fig. 3).

#### Coltivirus

**Fig. 4:**
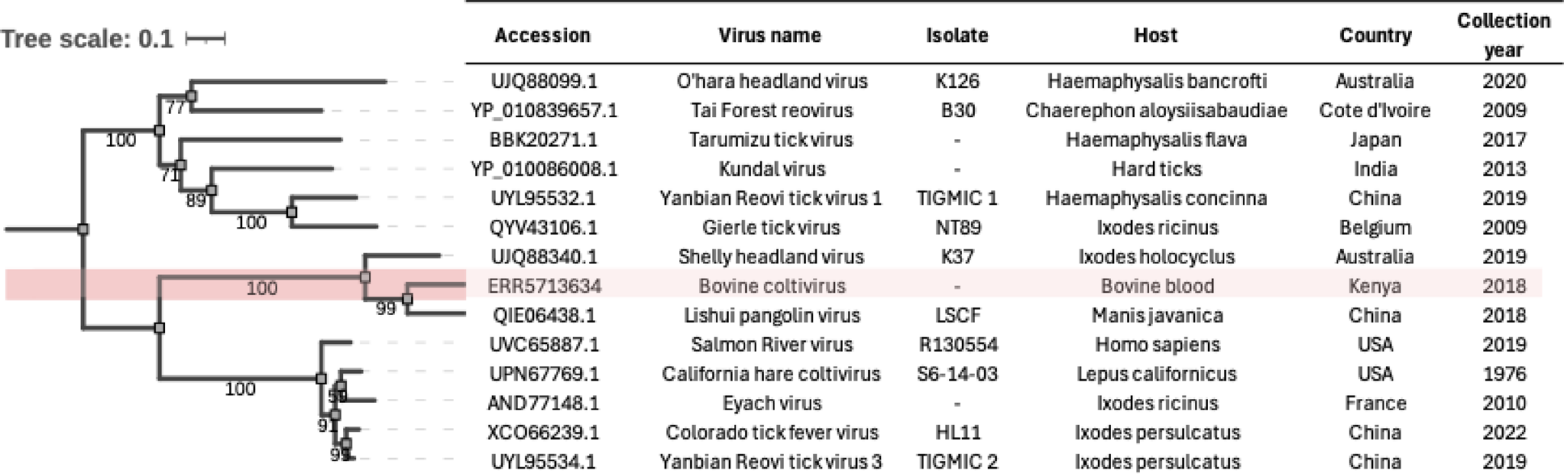
Midpoint rooted, maximum likelihood phylogenetic tree of the translated nucleotide sequences encoding for the coltivirus RdRp partial sequence (substitution model: LG + I + G4). The shaded branch indicates the sequence obtained for this study.

Partial genomic segments of a member of the genus *Coltivirus* were identified in one sample (ERR5713634, Dataset 2, Kenya). Coltiviruses are double-stranded, segmented RNA (dsRNA) viruses belonging to the family *Spinareoviridae*. The assembled contigs included sequences from six viral segments (VP3, VP4, VP5, VP7, VP9, and RdRp). Phylogenetic analysis placed the virus within the Coltivirus genus, clustering closely with Lishui pangolin virus (China) and Shelly Headlands virus (Australia; Fig. 4). Based on the genetic distance of segment VP7 with known species, the sequence is considered a new species within the Coltivirus genus (Table 1).

This virus was not identified in Data set 1 or the remaining transcriptomes from Data set 2.

**Table 1:** Partial coltivirus segments assembled from sample ERR5713634 and their similarities to the closest available published viral references.

Contig length (nucleotide)	Segment	Closest ncbi reference-NCBI accession (protein)	Closest reference virus name	aa identity (%)	Query cover (%)	Reference length
2901	VP3	QIE06440.1	Lishui pangolin virus	0.5679	0.99	3571
633	VP3	QIE06440.1	Lishui pangolin virus	0.4925	0.94	3571
1474	VP4	QIE06441.1	Lishui pangolin virus	0.6019	0.98	3152
1453	VP4	QIE06441.1	Lishui pangolin virus	0.449	0.99	3152
2266	VP5	QIE06442.1	Lishui pangolin virus	0.4723	0.89	2466
2264	VP7	UJQ88365.1	Shelly headland virus	0.3018*	0.46	1745
2001	VP9a	QIE06444.1	Lishui pangolin virus	0.3933	0.48	2040
990	RdRp	QIE06438.1	Lishui pangolin virus	0.8267	0.99	4345

#### Ephemeroviruses

**Fig. 5:**
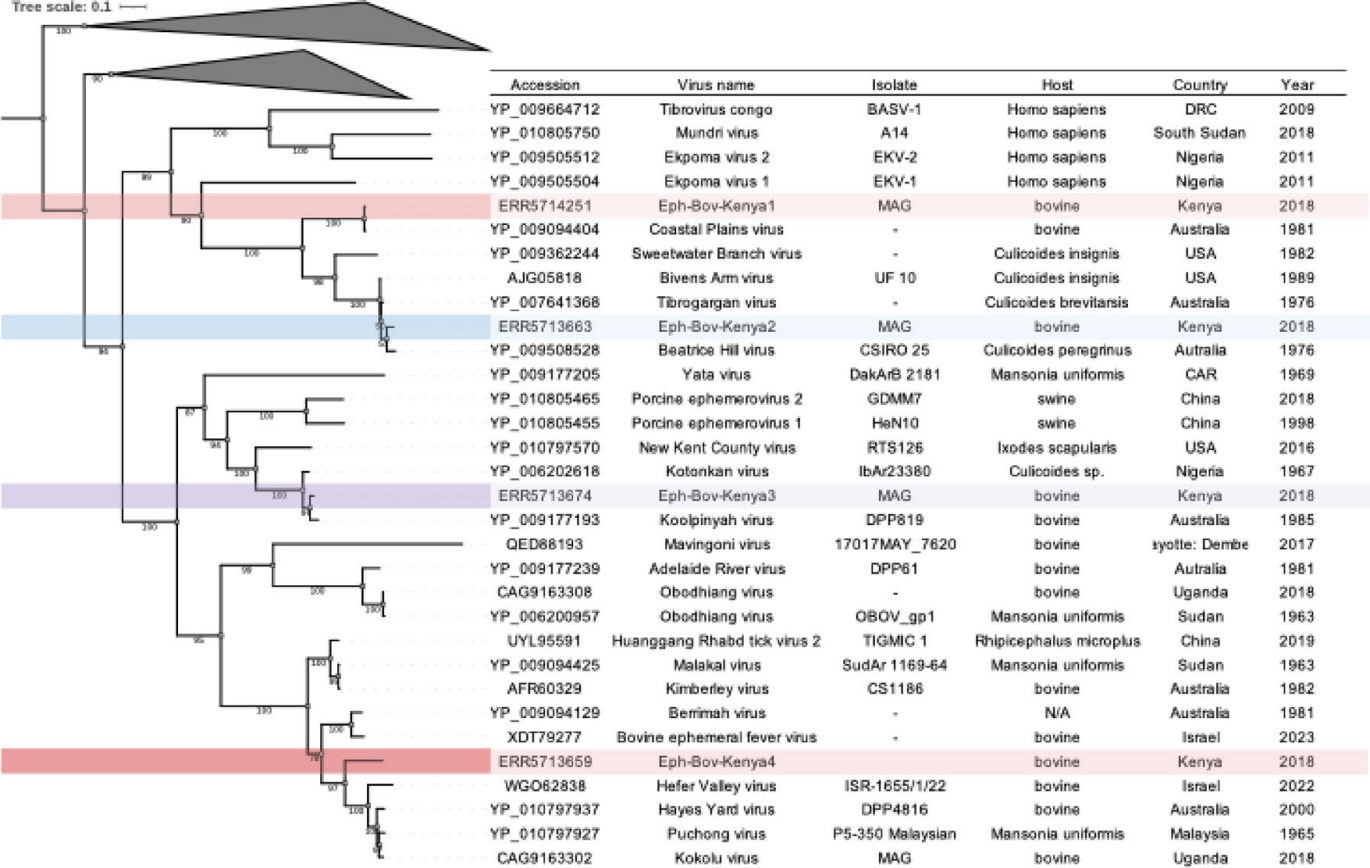
Maximum likelihood phylogenetic tree (midpoint rooted; substitution model: Q.insect + I + G4) of the translated nucleotide sequences encoding for the nucleoprotein gene coding region of Ephemeroviruses. Shaded branches indicate viral sequences obtained for this study. The shaded branches indicate viruses assembled for this study.

Four different ephemeroviruses were detected in Data set 2 (i.e., Kenyan cattle exposed to Theileria parva). Ephemeroviruses are negative-sense single-stranded RNA (-)ssRNA viruses from the family *Rhabdoviridae*. A complete ORF was assembled from SRA: ERR5713659, clustering with Hefer Valley (Israel), Hayes Yard (Australia), Kokolu (Uganda), and Puchong (Malaysia) viruses (Fig. 5). Partial genomes of different ephemeroviruses species were identified in transcriptomes with SRA accession numbers: ERR5713674: Clustered with Kotonkan (Africa) and Koolpinyah (Australia) ephemeroviruses; ERR5714251: Closely related to Coastal Plains virus; and ERR5713663: Grouped with Tirobragargan, Beatrice Hill, and Bivens Arm viruses (Fig. 5).

No sequences were detected in Data set 1 or other Australian samples in Data set 2.

### Other viruses

Other viruses identified by partial sequences (i.e., *de novo* assembled contigs, which were then queried against the NCBI nt viral database), were Bovine Foamy virus, for which the complete genome of proviral DNA of this species is 12k. We assembled eight > 400 nt long contigs ranging between 486 and 1265. All were > 99% similar to the closest Bovine foamy virus reference with a > 99% query cover. The contigs mapped partially to gag, pol and env coding regions. Fifty libraries from the published Australian transcriptome and 6 libraries from dataset 1 contained reads that mapped to Bovine Foamy virus, while no reads were mapped to the Kenyan bovine published transcriptomes.

**Table 2:** Summary of the main characteristics of the viruses detected in this study, including their family classification, known or suspected vectors, target cells, and virus infection progression.

Virus	Virus type-family	Transmission	Target cells	Virus infection progression
Bovine viral diarrhea virus 1	(-)ssRNA-*Flaviviridae*	direct	WBC; mucosa, etc.	acute - persistent
Bovine gammaherpesvirus 6	dsDNA *Orthoherpesviridae*	direct	WBC	chronic
Bovine foamy virus	*Retroviridae*	direct	monocytes, macrophages, fibroblasts	chronic-non pathogenic
Bovine hepacivirus	(-)ssRNA-*Flaviviridae*	Unknown (possibly vector tick)	Hepatocytes, possibly lymphocytes.	chronic
Coltivirus	dsRNA segmented- *Spinareoviridae*	Vector (tick)	Erythroid precursor cells (bone marrow), mature RBC, endothelial cells, CNS	acute
Ephemerovirus (e.g.) Bovine Ephemeral Fever (BEF)	(-)ssRNA- *Rhabdovirdae*	vector (mosquitoes/culicoides)	endothelial cells, macrophages and monocytes	acute (BEF)

## Discussion

While viral diversity in blood is more limited compared to other anatomical sites, we identified representatives of several important viral families—*Flaviviridae*, *Orthoherpesviridae*, *Spinareoviridae*, and *Rhabdoviridae*—in bovine blood transcriptomes. The viruses identified in the blood through RNA sequencing, are those with the ability to infect blood cells. Below, we discuss the viruses identified in this study.

### Viruses transmitted by direct contact

Three non-vector transmitted viruses were identified in the bovine viromes: Bovine viral diarrhea virus, Bovine gammaherpesvirus 6 and Bovine foamy virus. All three viruses can establish persistent infections in white blood cells. Of these, Bovine viral diarrhea virus (a pestivirus) is the most well-characterized.

In Data set 1, a sample from a mastitic cow exhibited high abundance of RNA that mapped to the genome of BoGHV6 a dsDNA virus. Bovine gammaherpesvirus 6, also known as bovine lymphotropic virus, is part of the *Gammaherpesvirinae* subfamily (which includes human pathogens like Epstein-Barr virus). Like other Gammaherpesviruses, BoGHV6 establishes latency in immune cells and can persist subclinical for years^37^. BoGHV6 is highly prevalent in cattle populations worldwide and spreads horizontally through bodily fluids. Its association with disease remains uncertain. While some studies have suggested possible links with pulmonary disease or abortion, most have found no consistent evidence of disease causation. At present, BoHV-6 is generally regarded as a commensal virus, and no specific factors required for pathogenicity have been established. Its role, if any, is most likely as a co-factor within complex disease syndromes, rather than as a primary pathogen^38^.

Interestingly, 50 libraries from the published Australian transcriptome (Data set 2) and six libraries from our study (Data set 1) contained reads mapping to Bovine Foamy Virus, while no reads were detected in the Kenyan bovine transcriptomes (Data set 2). Foamy viruses are complex retroviruses known to infect primates, cats, horses and cattle. Foamy viruses can establish lifelong infections, however, their impact on bovine health is generally regarded as negligible. Although this study could not determine whether the virus is endogenous or exogenous, previous reports indicate that bovine foamy virus can persist latently in blood without evidence of clinical disease^39,40^.

### Vector-borne viruses

The following vector-borne viral genera were identified in this study: hepaciviruses, ephemeroviruses and coltiviruses.

Bovine hepacivirus was detected in both healthy and mastitic cows from Data set 1 but was not found in any of the published transcriptomes in Data set 2. Hepaciviruses are known to infect humans, dogs, horses, and cattle. While these viruses have a well-established pathogenic role in humans, their association with disease in cattle remains unclear^41,42^. Beyond their primary replication in hepatocytes, hepaciviruses can also infect lymphocytes and monocytes. Human Hepatitis C virus has been shown to bind to the surface of red blood cell, which may contribute to persistence in circulation^43^. Similarly, bovine hepacivirus RNA has been detected in cattle serum persisting for over six months, consistent with the ability to establish persistent infections. Despite this persistence and evidence of strong liver tropism, infected animals did not show clinical signs of disease, alterations in liver enzyme levels, or histological evidence of liver damage at postmortem examination^41^. The route of bovine hepacivirus transmission remains unknown, but its detection in ticks, suggests a possible vector-borne transmission pathway^44^.

Multiple ephemeroviruses species were detected in Kenyan samples (Dataset 2), but none were identified in the Australian transcriptomes from either Datasets 1 or 2. The complete ORF or partial sequences of these viruses were recovered from four animals, three of which later died after exposure to *Theileria parva*, according to the original study (Dataset 2).

Ephemeroviruses are transmitted by hematophagous arthropods and have only been detected in cattle, water buffalo, pigs and deer. These viruses are widespread in tropical and subtropical regions, including Africa, Australia, the Middle East and Asia^45–47^. The most well-known virus in this group is Bovine Ephemeral Fever Virus (BEFV), which is likely transmitted by haematophagous insects^45^. However, ephemeroviruses such as New Kent County virus and Huanggang rhabd tick virus 2 have been identified in ticks in the US and China, suggesting a broader range of potential vectors.

While BEFV may be controlled through vaccination, other ephemeroviruses may be overlooked as contributors to febrile illnesses in cattle^48^. These viruses often cause transient clinical signs but can modulate immune function, leading to temporary immune suppression. In regions where ephemerovirus vectors are prevalent, genomic surveillance to catalogue the species diversity, combined with targeted diagnostic techniques, is essential for identifying potential pathogenic strains and understanding their impact on cattle health.

A previously uncharacterized coltivirus species was identified in one sample from the Kenyan study (Dataset 2). Recognised members of the genus *Coltivirus* include the Colorado tick fever virus and Eyach Virus. Colorado Tick viruses is transmitted by ticks (*Dermacentor* spp.) with chipmunks, squirrels and other small mammals serving as primary reservoirs, while humans and other mammals are considered accidental hosts^49^. Eyach Virus, on the other hand, is associated with tick-borne encephalitis, with the European rabbit suspected to be its main reservoir^50^. To date, there is one previous report from a coltivirus collected from bovine blood in Africa (Uganda)^51^, and most related viruses have been identified exclusively through shotgun metagenomic sequencing^52^. So, their host range, transmission dynamics and potential pathogenicity remains largely unstudied.

Only 20 of the 70 processed blood samples passed RNA quality thresholds for sequencing, which may reflect the inherent difficulty of extracting sufficient RNA from blood, particularly when WBC counts are low. This may have introduced a potential bias against detecting viruses that significantly affect WBC populations, and may therefore have limited the diversity of the virome captured in this dataset. Additionally, as this study employed a metatranscriptomic approach targeting total RNA, there are important considerations when interpreting the detection and relative abundance of RNA versus DNA viruses. For example, the detection of a herpesvirus at high abundance in one sample raises the question of whether this reflects active viral transcription or the presence of residual viral DNA that was not fully depleted during library preparation. While the RiboZero Plus kit removes ribosomal RNA and partially depletes host DNA, it does not eliminate all DNA contaminants, particularly from high-titer DNA viruses. Consequently, our approach is inherently more sensitive to RNA viruses and may underrepresent DNA viruses unless they are transcriptionally active or present in high DNA copy numbers. These factors must be considered when interpreting both the breadth and abundance of viruses detected in blood transcriptomes using RNA-based methods.

In addition, the relatively small number of viruses identified across the larger dataset likely reflects both biological and technical factors. Biologically, blood is not a compartment where high viral diversity is typically expected, in contrast, for example, to respiratory or enteric samples. Technically, variation in sample processing and library preparation methods across studies, for example, whole blood versus WBC extraction, or different library preparation approaches, further influences sensitivity. These findings illustrate both the opportunities and limitations of leveraging existing metatranscriptomic datasets: while they enable broad, opportunistic surveys of the blood virome, differences in study design and sample quality inevitably shape the virome profiles that can be recovered.

In summary, this study contributes to understanding the diversity of the bovine virome, which can be acquired through different transmission mechanisms and have diverse host cell tropism and disease progression patterns. The results presented here reflect a snapshot of the local bovine virome at a single time point in each sampling location. However, viral infections may be influenced by the sporadic presence of competent arthropod vectors, changing population demographics, their previous exposure to disease, and the infection status of individuals at the time of sampling. Therefore, the viral diversity observed in this study represents only a fraction of the global bovine blood virome, underscoring the need for comprehensive and sustained surveillance to fully understand its true diversity.

By leveraging blood and white blood cell transcriptomes from studies conducted for unrelated purposes, this study demonstrates the utility of metatranscriptomics in passive viral surveillance. Further studies are required to determine the clinical significance of these viruses. Understanding their epidemiology, impact on cattle health, and potential economic consequences will help identify which viruses warrant targeted control strategies.

## Supplementary Information

Below is the link to the electronic supplementary material.

Supplementary Material 1

## Data Availability

Raw data generated in this study are available in NCBI BioProject PRJNA1250162. Published datasets analysed include BioProjects PRJEB44244, PRJNA917329, PRJNA305942, PRJNA392196, and PRJNA616134. Newly identified viral sequences were submitted to GenBank under accession numbers PV578941-PV578943 and BK071684-96.
