# Supplementary material for: Leveraging metatranscriptomics for the characterisation of bovine blood viromes
Source: Sci Rep. 2025 Oct 21;15:36670. doi: 10.1038/s41598-025-20367-1 (PMC12540862; doi:10.1038/s41598-025-20367-1)
Supplement: Supplementary file 1 — Supplementary Material 1 [file 41598_2025_20367_MOESM1_ESM.docx]

**Leveraging metatranscriptomics for the characterisation of bovine blood viromes**

Barbara Brito^1,2^, Matthew DeMaere ^2^, Ian Lean^3^, Mark Hazelton^1^, Brendon A O’Rourke^1^, Edward C Holmes^4^, John K House^5^, Sam Rowe^5^, Garry Myers^2^ and Piklu Roy Chowdhury^2^.

^1^ New South Wales Department of Primary Industries and Regional Development (DPIRD), Elizabeth Macarthur Agricultural Institute, Menangle, NSW, Australia

^2^ Australian Institute for Microbiology and Infection, University of Technology Sydney, Ultimo, NSW, Australia

^3^ Scibus, Camden, NSW 2570, Australia

^4^ School of Medical Sciences, The University of Sydney, New South Wales, Australia.

^5^ Faculty of Science, Sydney School of Veterinary Science, The University of Sydney, Camden, New South Wales 2570, Australia.

Table S1- Sample processing of published sequences:

| Bioproject (# Biosamples) | Title | Animal and collection | Sample processing | Extraction | Library Preparation | Sequencing |  |
| --- | --- | --- | --- | --- | --- | --- | --- |
| PRJNA616134 (10) | Gene expression of the heat stress response in bovine peripheral white blood cells and milk somatic cells in vivo | Data from 12 non-pregnant multiparous Holstein Fresian cows | Centrifuged at 4C, fractionation and WBC stabilization- stored in RNAlater | RiboPuer Blood Kit (Ambion) | SureSelect Strand Specific RNA Library Prep Kit (Agilent) mRNA | HiSeq 3000 (Illumina Inc) in a 150-cycle paired-end run |  |
| PRJNA616134  PRJNA917329 (198) PRJNA305942 (118) PRJNA392196 (6) | Gene expression and RNA splicing explain large proportions of the heritability for complex traits in cattle | 382 lactating (Holstein and Jersey) | Centrifuged at 4C, fractionation and WBC stabilization- stored in RNAlater | RiboPuer Blood Kit (Ambion) | SureSelect Strand Specific RNA Library Prep Kit (Agilent) mRNA | HiSeq3000 (Illumina Inc) or NovaSeq6000 (Illumina Inc) genome analyzer in a paired-end, 150-cycle run | |
| PRJEB44244 (245) | Longitudinal study of blood-derived transcriptomes of Boran cattle naturally exposed to Theileria parva | 30 Boran cattle from the Kapiti research station in Machakos county, Kenya. | Tubes were then centrifuged at 300 g for 5 min at RT and the supernatant discarded. The pellet was rinsed twice with 15 ml of PBS + centrifuged, resuspended in tri-reagent | RNA was extracted by phenol chloroform | TruSeq Stranded mRNA | Illumina HiSeq | |

Supplementary table S2: Data output analysed after QC trimming per library. Total RNA sequencing from blood samples of cows with and without mastitis. Twenty out of 70 samples that had a DV>200 were sequenced (BioProject: PRJNA1250162).

| Library | Reads after QC (%) | | Bases after QC (%) | | Status | BioSamples |
| --- | --- | --- | --- | --- | --- | --- |
| BM_06 | 126334140 | (99.93%) | 18069685232 | (95.29%) | Mastitis | SAMN47932226 |
| BM_11 | 128869756 | (99.96%) | 18290493384 | (94.58%) | Mastitis | SAMN47932227 |
| BM_12 | 103141786 | (99.93%) | 14297261968 | (92.34%) | Mastitis | SAMN47932228 |
| BM_13 | 125288824 | (99.97%) | 17654855197 | (93.91%) | Mastitis | SAMN47932229 |
| BM_17 | 138282820 | (99.95%) | 19607916235 | (94.48%) | Mastitis | SAMN47932230 |
| BM_19 | 151804270 | (99.94%) | 21393920325 | (93.9%) | Mastitis | SAMN47932231 |
| BM_22 | 111685978 | (99.95%) | 15938154735 | (95.09%) | Mastitis | SAMN47932232 |
| BM_26 | 116405556 | (99.96%) | 16316066744 | (93.41%) | Mastitis | SAMN47932233 |
| BM_27 | 139875272 | (99.92%) | 19504005367 | (92.88%) | Mastitis | SAMN47932234 |
| BM_28 | 162814936 | (99.93%) | 23002170333 | (94.12%) | Mastitis | SAMN47932235 |
| BM_29 | 107102218 | (99.66%) | 14624550324 | (90.72%) | Mastitis | SAMN47932236 |
| BM_35 | 120298186 | (99.94%) | 17136193810 | (94.91%) | Mastitis | SAMN47932237 |
| BM_37 | 99991674 | (99.9%) | 14038189285 | (93.5%) | No Mastitis | SAMN47932238 |
| BM_41 | 126270368 | (99.96%) | 17878307544 | (94.35%) | No Mastitis | SAMN47932239 |
| BM_49 | 124919934 | (99.97%) | 17828271943 | (95.12%) | No Mastitis | SAMN47932240 |
| BM_60 | 133608282 | (99.97%) | 18991419907 | (94.73%) | No Mastitis | SAMN47932241 |
| BM_64 | 162893752 | (99.92%) | 23187865410 | (94.82%) | No Mastitis | SAMN47932242 |
| BM_67 | 127349912 | (99.95%) | 18098611703 | (94.7%) | No Mastitis | SAMN47932243 |
| BM_68 | 130867296 | (99.95%) | 18561407149 | (94.51%) | No Mastitis | SAMN47932244 |
| BM_69 | 128629238 | (99.93%) | 18353711613 | (95.05%) | No Mastitis | SAMN47932245 |

Supplementary table S3: NCBI GenBank’s accession numbers of assembled viruses.

| Sequence ID | Source | Collection data | Country | Host | BioProject | BioSample | SRA | Accession number |
| --- | --- | --- | --- | --- | --- | --- | --- | --- |
| BVDV1/bovine/ERR5713674/Kenya/2018 | bovine blood | 2018 | Kenya | Bovine | PRJEB44244 | SAMEA8556471 | ERR5713634 | BK071688 |
| BVDV1/bovine/26/Australia/2021 | bovine blood | Mar-21 | Australia | Bovine | PRJNA1250162 | SAMN47932233 | SRS24720467 | PV578941 |
| Coltivirus/bovine/Kenya/2018/1125_VP3 | bovine blood | 2018 | Kenya | Bovine | PRJEB44244 | SAMEA8556471 | ERR5713634 | BK071689 |
| Coltivirus/bovine/Kenya/2018/1125_VP3.1 | bovine blood | 2018 | Kenya | Bovine | PRJEB44244 | SAMEA8556471 | ERR5713634 | BK071690 |
| Coltivirus/bovine/Kenya/2018/1125_VP4 | bovine blood | 2018 | Kenya | Bovine | PRJEB44244 | SAMEA8556471 | ERR5713634 | BK071691 |
| Coltivirus/bovine/Kenya/2018/1125_VP4.1 | bovine blood | 2018 | Kenya | Bovine | PRJEB44244 | SAMEA8556471 | ERR5713634 | BK071692 |
| Coltivirus/bovine/Kenya/2018/1125_VP5 | bovine blood | 2018 | Kenya | Bovine | PRJEB44244 | SAMEA8556471 | ERR5713634 | BK071693 |
| Coltivirus/bovine/Kenya/2018/1125_VP7 | bovine blood | 2018 | Kenya | Bovine | PRJEB44244 | SAMEA8556471 | ERR5713634 | BK071694 |
| Coltivirus/bovine/Kenya/2018/1125_VP9a | bovine blood | 2018 | Kenya | Bovine | PRJEB44244 | SAMEA8556471 | ERR5713634 | BK071695 |
| Coltivirus/bovine/Kenya/2018/1125_RdRp | bovine blood | 2018 | Kenya | Bovine | PRJEB44244 | SAMEA8556471 | ERR5713634 | BK071696 |
| Ephemerovirus_1125/bovine/Kenya/2018 | bovine blood | 2018 | Kenya | Bovine | PRJEB44244 | SAMEA8556471 | ERR5713674 | BK071684 |
| Ephemerovirus_1113/bovine/Kenya/2018 | bovine blood | 2018 | Kenya | Bovine | PRJEB44244 | SAMEA8556461 | ERR5713663 | BK071685 |
| Ephemerovirus_1110/bovine/Kenya/2018 | bovine blood | 2018 | Kenya | Bovine | PRJEB44244 | SAMEA8556458 | ERR5714251 | BK071686 |
| Ephemerovirus_1117/bovine/Kenya/2018 | bovine blood | 2018 | Kenya | Bovine | PRJEB44244 | SAMEA8556465 | ERR5713659 | BK071687 |
| Hepacivirus_68/bovine/Australia/2021 | bovine blood | Mar-21 | Australia | Bovine | PRJNA1250162 | SAMN47932244 | SRS24720462 | PV578943 |
| Hepacivirus_19/bovine/Australia/2021 | bovine blood | Mar-21 | Australia | Bovine | PRJNA1250162 | SAMN47932231 | SRS24720464 | PV578942 |
